# Psychiatric polypharmacy in adult patients at the only referral psychiatric institute in Botswana: A cross-sectional study

**DOI:** 10.1371/journal.pone.0359278

**Published:** 2026-09-25

**Authors:** Boikhutso Changu Kinsman, Hlanganiso Roy, Anthony A. Olashore

**Affiliations:** Department of Psychiatry, Faculty of Medicine, University of Botswana, Gaborone, Botswana; National Taichung University of Science and Technology, TAIWAN

## Abstract

**Background:**

Despite growing acceptance in some clinical settings, its risks necessitate caution due to adverse effects, drug interactions, and poor adherence. We aimed to find the prevalence, patterns, and predictors of psychiatric polypharmacy among adults at Sbrana Psychiatric Hospital (SPH) in Lobatse, Botswana.

**Methods:**

This cross-sectional study involved the collection of demographic and clinical data via interviews and medical records from 300 participants at SPH, which were subsequently analyzed using multivariable logistic regression.

**Results:**

The mean age of participants was 36.5 years (SD 12.1), and they were predominantly male (55.3%). The overall prevalence of psychiatric polypharmacy was 49.7%, with the most prevalent subtype being multi-class polypharmacy (79.2%). Inpatients (Adjusted Odds Ratio [AOR] = 5.00; 95% Confidence Interval [CI]: 2.70,9.25), individuals with substance use disorder (AOR = 4.60; 95% CI: 1.18, 1.18,17.86), and those diagnosed with bipolar mood disorder (AOR = 2.67; 95% CI: 1.49,4.80) exhibited significant associations with psychiatric polypharmacy.

**Conclusions:**

Psychiatric polypharmacy is common in this group, mostly due to bipolar disorder or substance use disorders. It is crucial to identify high-risk patients for reviews and to train clinicians in rational prescribing to improve medication use and minimize risks in Botswana.

## Introduction

While not disregarding evidence supporting its use and its effectiveness in certain clinical scenarios, psychiatric polypharmacy remains an essential area of concern that merits focused attention, particularly considering its association with adverse outcomes that can adversely affect patients’ quality of life [1]. It involves the use of two or more psychotropics simultaneously to manage mental health conditions in a patient at the same time [2].

The prevalence of psychiatric polypharmacy has been estimated to range between 13% and 90% worldwide; the prevalence varies significantly across regions, specific psychiatric disorders, and all age groups [3–7]. A systematic review conducted among patients with bipolar mood disorder reported a psychiatric polypharmacy prevalence rate of 32.7% [8]. Among participants with a diagnosis of intellectual disability in the United Kingdom, one study found that 23% were on psychiatric polypharmacy, while two other studies conducted in Europe among patients with schizophrenia and dementia found prevalence rates of 71% and 25.3%, respectively [9–11]. Studies conducted in Africa have also reported significant prevalence rates of psychiatric polypharmacy of 83.45%, 66.0%, and 93.8% in Ethiopia, Ghana, and South Africa, respectively [12–14].

Studies have provided insights into the various types of psychiatric polypharmacy as classified by the National Association of State Mental Health Program Directors (NASMHPD). Previous research in South Asia, Europe, and West Asia identified multi-class polypharmacy as the most prevalent type [15–17]. Multi-class polypharmacy is when a patient is prescribed medications that belong to different classes to treat their symptoms [18,19]. On the contrary, a study conducted in Africa found that adjunctive polypharmacy, which involves the use of one medication to mitigate the side effects caused by another medication from a different class [20], was the most common type of psychiatric polypharmacy among participants [21]. Other types include same-class polypharmacy, which involves using multiple psychiatric medications that belong to the same class, and augmentation polypharmacy, which is the use of a medication at a lower-than-usual dose alongside another medication from a different class in full therapeutic doses to treat the same symptoms [3,19,21]. Augmentation polypharmacy can also mean prescribing a medication that would usually not be used alone to treat symptoms, for example, prescribing lithium to augment antidepressants [16]. When compared to the other types, same-class psychiatric polypharmacy is associated with increased risks of drug interactions and side effects [3,22,23].

Treatment guidelines recommend starting with a single medication as the first-line approach, owing to its simplicity in regimens and lower likelihood of adverse effects [21,24]. Nevertheless, the persistent and multifaceted nature of mental health disorders often necessitates the use of multiple psychotropic medications [15,25–27].

The phenomenon of psychiatric polypharmacy is driven by a variety of factors, ranging from patient characteristics, illness characteristics, and prescriber characteristics [3,6,7]. Patient factors such as age, gender, and marital status, as well as illness characteristics such as having a substance use history and a longer duration of hospitalization, were found to be significantly associated with psychiatric polypharmacy in earlier studies [18,28–39]. Severe or treatment-resistant mental health disorders, such as schizophrenia and bipolar mood disorder, are more likely to require multiple medications to manage persistent or refractory symptoms. Furthermore, previous studies have established that the likelihood of psychiatric polypharmacy is influenced by the diagnosis [4,40–42]. Among the illness characteristics, research has demonstrated that the incidence of psychiatric polypharmacy is generally elevated in patients with multiple psychiatric disorders [43–45]. Existing research that has investigated prescriber characteristics has reported that having multiple prescribers or having a psychiatrist as a sole prescriber was found to be associated with psychotropic polypharmacy [1,46].

The use of multiple medications leads to unnecessary healthcare expenditure, heightens the risk of adverse side effects, leads to drug interactions, and, consequently, treatment discontinuation or reduced patient adherence; these may eventually compromise the overall effectiveness of the treatment and worsen clinical outcomes [9,47–51]. Older patients are particularly more susceptible to these adverse drug reactions associated with psychiatric polypharmacy because of changes in pharmacokinetics associated with aging [52–54]. In light of its negative outcomes, a study in the United States assessed the efficacy of polypharmacy versus monotherapy; the study compared patients discharged on monotherapy with those discharged on polypharmacy and found that polypharmacy did not reduce the risk of re-admission or relapse, emphasizing that the benefits of polypharmacy remain controversial [55].

Despite the global focus on psychiatric polypharmacy and its association with negative outcomes, there is a paucity of local data on the prevalence, determinants, and outcomes of psychiatric polypharmacy in adult psychiatric patients in Botswana. Botswana’s health care context, characterized by inadequate resources that limit service delivery, presents unique considerations that may not be adequately addressed in existing global literature, emphasizing the importance of generating localized insights [56]. Existing research on psychiatric polypharmacy in Botswana has been limited, with previous studies focusing exclusively on children and adolescents [57]. While the study’s findings offered valuable insights into the prevalence and predictors of polypharmacy in the age group, their applicability to the adult population remains unclear. Additionally, the prior study indicated a substantial prevalence of psychiatric polypharmacy among children and adolescents [57], which may imply a heightened burden within the adult population, especially since polypharmacy increases with older age. Although there is no published data to substantiate this, reports from Botswana’s sole referral psychiatric institute indicate a substantial prevalence of severe psychiatric conditions like schizophrenia and bipolar disorder. These conditions are frequently treated with multiple psychotropic drugs aimed at various symptoms or mechanisms, resulting in psychiatric polypharmacy [52,58–60].

This gap in knowledge limits the ability to assess whether current prescribing practices align with best practices or contribute to adverse outcomes and hinders the development of effective interventions to optimize medication management in psychiatric care. Therefore, our study aimed to ascertain the prevalence, patterns, and associated factors of psychiatric polypharmacy among adult patients at Sbrana Psychiatric Hospital. This study’s findings may provide valuable insights to guide policy development, inform clinical decision-making, and promote safer and more effective medication practices.

## Methods and materials

### Study design, site, and population

The study was conducted at Sbrana Psychiatric Hospital, the country’s sole referral psychiatric facility, located in Lobatse in the southeastern district of Botswana, using a cross-sectional research design. It accepts referrals from private facilities and other government-owned community and primary centers across the country. The hospital staff comprises multiple disciplines, including psychiatrists, psychologists, social workers, occupational therapists, and nurses.

The study included inpatients and outpatients who were 18 years old or older, had a DSM-5-TR psychiatric diagnosis, were receiving pharmacotherapy, and were able to communicate in either English or Setswana. Patients who were acutely ill or could not provide informed consent were excluded from the study.

### Sample size determination

The Cochran formula was used to calculate the sample size, using the probability of an outcome occurring (83.45%), derived from a similar study [41].


$$\begin{array}{c} N=Z^{2}\frac{\left(p\left(1-p\right)\right)}{d^{2}} \end{array}$$

Where: N = estimated minimum sample size, Z = Standard normal distribution 1.96, which corresponds to a 95% confidence interval, p = probability of an outcome occurring, which was 83.45%, d = level of precision at 0.05 (5%), Consequently, N = 212.

To adjust for no response, the formula below was used


$$\begin{array}{c} N2=\left(\frac{1}{\left(1-NR\right)}\cdot N1\right) \end{array}$$

Where: N2 = adjusted sample size, N1 = initial sample size (212), NR = estimate of proportion of no response (20%), hence adjusted sample size (N2) = 266. However, the final sample size was increased to 300 to increase the study’s power.

### Sampling method

A stratified random sampling method was used for the present study. Care mode was used to stratify participants as outpatients or inpatients. With a sample size of 300, equal allocation was performed across strata, with 150 participants in each. Simple random sampling was then conducted within each group. For simple random sampling, a list of patients was used to assign random numbers to participants; the corresponding numbers were written on equal-sized pieces of paper, which were then placed in a container and thoroughly mixed. Numbers were drawn at random until the required number of participants was reached. This method was chosen to ensure adequate representation of both inpatients and outpatients in the sample.

### Data collection procedure

Participants who met the eligibility criteria were drawn from outpatients and inpatients; both were assessed on different days, as convenient for them. As per the previously described sampling technique, selected participants were individually called to a consultation room, where they were briefed on the study. Participants willing to take part in the study were assured of confidentiality and informed of their right to withdraw at any time, for any reason, without consequences. The participants were then asked to provide written informed consent. Data were then collected from the consenting participants through interviews and review of their medical records. The participants were interviewed in their preferred language (English or Setswana), and the principal investigator recorded the responses to the questionnaires as narrated by the participants. Only those who consented in writing were included in the study. Participant recruitment and review of their medical records were conducted simultaneously over 6 months (from January 2024 to June 2024).

### Measures

#### Socio-demographic data questionnaire.

The questionnaire was developed after reviewing relevant literature. The socio-demographic information used in the questionnaire was obtained from interviews with participants and from a review of their medical records. Socio-demographic data included variables such as age (as determined by date of birth), sex, and relationship status. The questionnaire was developed in English, then translated into Setswana and back into English by two experts.

### Clinical data extraction tool

The tool was developed after a review of the relevant literature. Clinical data were collected from participants’ medical records. The clinical data included variables such as mode of care, psychiatric diagnosis, prescribing pattern, and the type of polypharmacy. Clinical data regarding the psychotropic medication prescribed for each participant in the sample were obtained from the last prescription written by the medical practitioner. The information used from the prescription included the total number of psychotropics prescribed, the doses, and the class of the medication.

Psychiatric polypharmacy was categorized into various types based on the National Association of State Mental Health Program Directors’ classification of psychotropic polypharmacy.

### DSM-5-TR

The DSM-5-TR, or the Diagnostic and Statistical Manual of Mental Disorders, Fifth Edition, Text Revision, is a comprehensive guide published by the American Psychiatric Association (APA) in March 2022. It is an updated version of the DSM-5, and it serves as a resource for diagnosing and classifying mental health disorders. The principal investigator used DSM-5-TR to assign psychiatric diagnoses to the participants, which was a required variable in the clinical data questionnaire. The principal investigator was a psychiatry resident and had experience in psychiatry. Participants who did not meet a diagnostic criterion as per DSM-5-TR were excluded from the study.

### Ethical considerations

Approval to conduct the study was obtained from the the University of Botswana’s Research and Ethics Committee (UBR/RES/IRB/BIO/GRAD/185), the Ministry of Health, and the hospital management of Sbrana Psychiatric Hospital.

All procedures carried out in studies involving human participants were conducted in accordance with the ethical standards of the institutional and national research committees, as well as with the 1964 Declaration of Helsinki and its subsequent amendments or equivalent ethical standards. All participants provided written informed consent prior to taking part in the study.

### Data analysis

The Statistical Package for the Social Sciences (SPSS), version 27, was used for the analysis. Descriptive statistics: frequencies and percentages were used for categorical socio-demographic and clinical variables, such as gender, relationship status, mode of care, and DSM-5-TR psychiatric diagnosis. Means and standard deviations were calculated for continuous variables, including age, duration of psychiatric treatment, and duration of current hospitalization. The choice of the independent variables was guided by the literature [18,28–39,42]. First, bivariate logistic regression was used to assess the association between the selected independent variables and the outcome: the presence or absence of psychotropic polypharmacy. All variables significant at p < 0.05 in the bivariate analysis were then entered into a multivariable model to control for confounding. We conducted the Omnibus Test of Model Coefficients and evaluated multicollinearity; tolerance values below 0.1 and Variance Inflation Factors (VIFs) exceeding 10 were considered indicators of high multicollinearity. The Hosmer-Lemeshow test was also performed to assess the goodness of fit of the logistic regression model; a p-value < 0.05 indicates a good fit. A single-step multivariable regression model was used to identify variables that predicted the outcome, and p-values < 0.05 were considered significant in this study.

## Results

### Socio-demographic and clinical characteristics of the participants

A total of 300 participants were included in the study; the mean age (SD) was 36.5 (12.1) years. Males were more represented than females at 166 (55.3%). The sample size of 300 was evenly distributed between outpatients and inpatients; 50.0% of the participants were outpatients and 50.0% were inpatients. A larger proportion of the participants were diagnosed with schizophrenia and other psychotic disorders, totaling 173 (57.7%). (Tables 1 and 2).

**Table 1:** Socio-demographic characteristics of 300 participants in Sbrana Psychiatric Hospital.

Variable	Statistics	
	N	Mean (SD)
**Age**	**300**	**36.5(12.1)**
	N	Percentage
**Gender**	**300**	**100**
Male	166	55.3
Female	134	44.7
**Relationship status**	**300**	**100**
In a committed relationship	103	34.3
Not in a relationship	197	65.7
**Level of education**	**300**	**100**
No education	14	4.7
Primary	33	11.0
Secondary	146	48.7
Tertiary	107	35.7
**Location of residence**	**300**	**100**
Rural	128	42.7
Urban	172	57.3
**Religion**	**300**	**100**
Christianity	253	84.3
No affiliation	39	13.0
Others^a^	8	2.7
**Employment status**	**300**	**100**
Employed	98	32.7
Unemployed	185	61.7
Student	17	5.7

^**a**^ **Others = African traditional religion, Islam**

**Table 2:** Clinical characteristics of 300 participants in Sbrana Psychiatric Hospital.

Variables	Statistics	
	N	Mean (SD)
Duration of psychiatric treatment (years) Duration of current hospitalization (months)	**300** **150** ^**a**^	**2.07(0.86)** **1.38(0.49)**
	N	Percentage
**Treatment setting**	**300**	**100**
Outpatient	150	50.0
Inpatient	150	50.0
**Primary Psychiatric diagnosis**	**300**	**100**
Schizophrenia and other psychotic disorders	173	57.7
Bipolar mood disorder Major depressive disorder	50 34	16.7 11.3
Trauma and stress related disorders	20	6.7
Substance use disorders	16	5.3
Others^b^	7	2.3
**Past psychiatric history**	**300**	**100**
Newly diagnosed patient	58	19.3
Known psychiatric patient	242	80.7
**Psychiatric co-morbidity**	**300**	**100**
Absent	229	76.3
Present	71	23.7
**Medical co-morbidity**	**300**	**100**
Absent	204	68.0
Present	96	32.0
**Previous admissions**	**300**	**100**
Yes	199	66.3
No	101	33.7
**Psychiatrist care given**	**300**	**100**
Yes No	127 173	42.3 57.7
**Type of intervention**	**300**	**100**
Only pharmacological	243	81.0
Pharmacological + psychotherapy	57	19.0

^**a**^ **N not equal to 300 due to outpatients.** ^**b**^**Others = Narcolepsy, somatic symptom disorder, ADHD, personality disorders.**

### Prevalence of psychiatric polypharmacy

**Fig 1:**
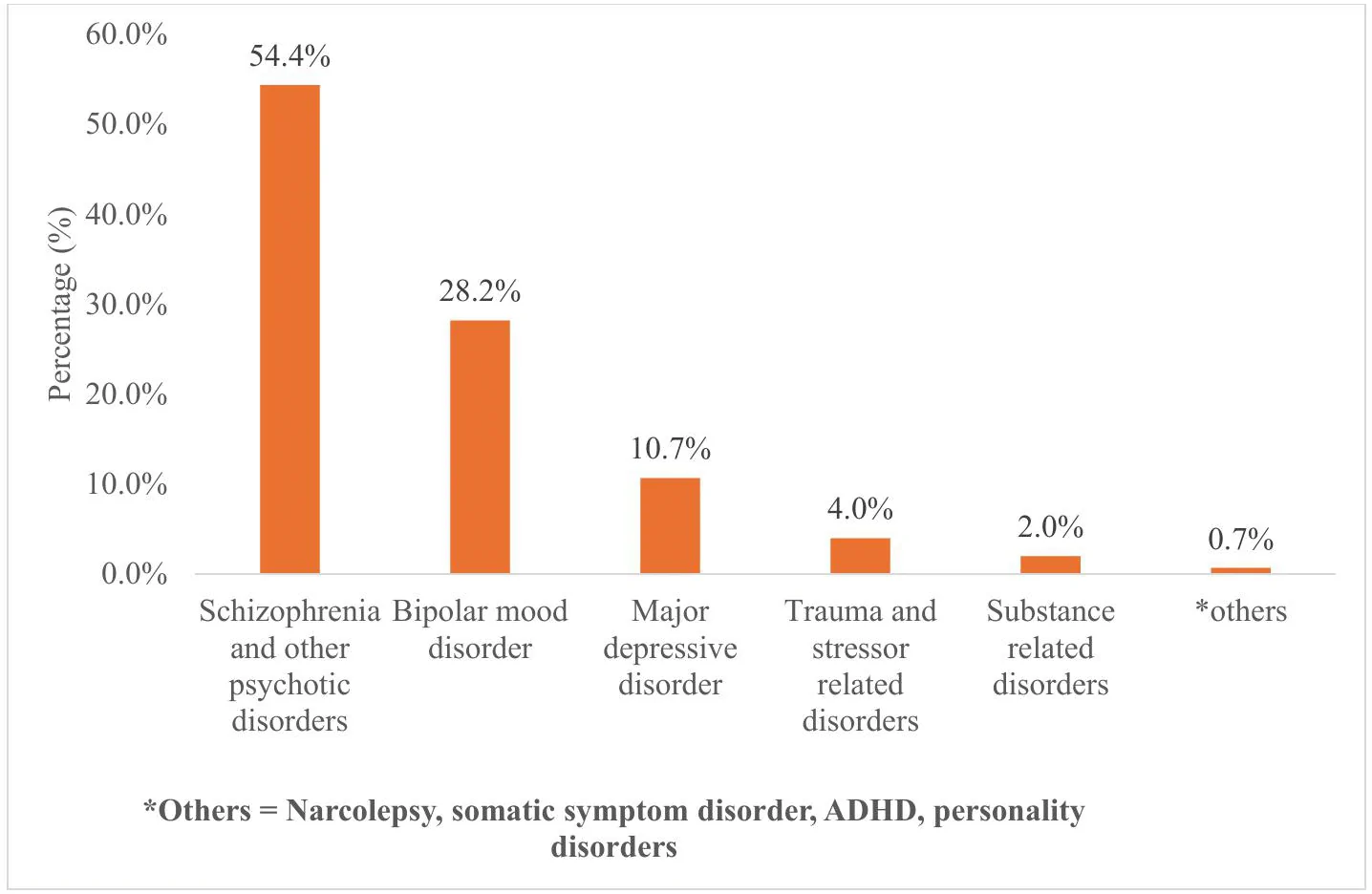
Prevalence of psychiatric polypharmacy by diagnosis among 300 participants in Sbrana Psychiatric Hospital.

Psychiatric polypharmacy was found in 49.7% of all the participants included. Most of the participants receiving psychiatric polypharmacy were males (55.7%). With regards to the psychiatric diagnosis, most of the participants with psychiatric polypharmacy had a diagnosis of schizophrenia and other psychotic disorders (54.4%) (Fig 1).

### Pattern of psychiatric polypharmacy

**Fig 2:**
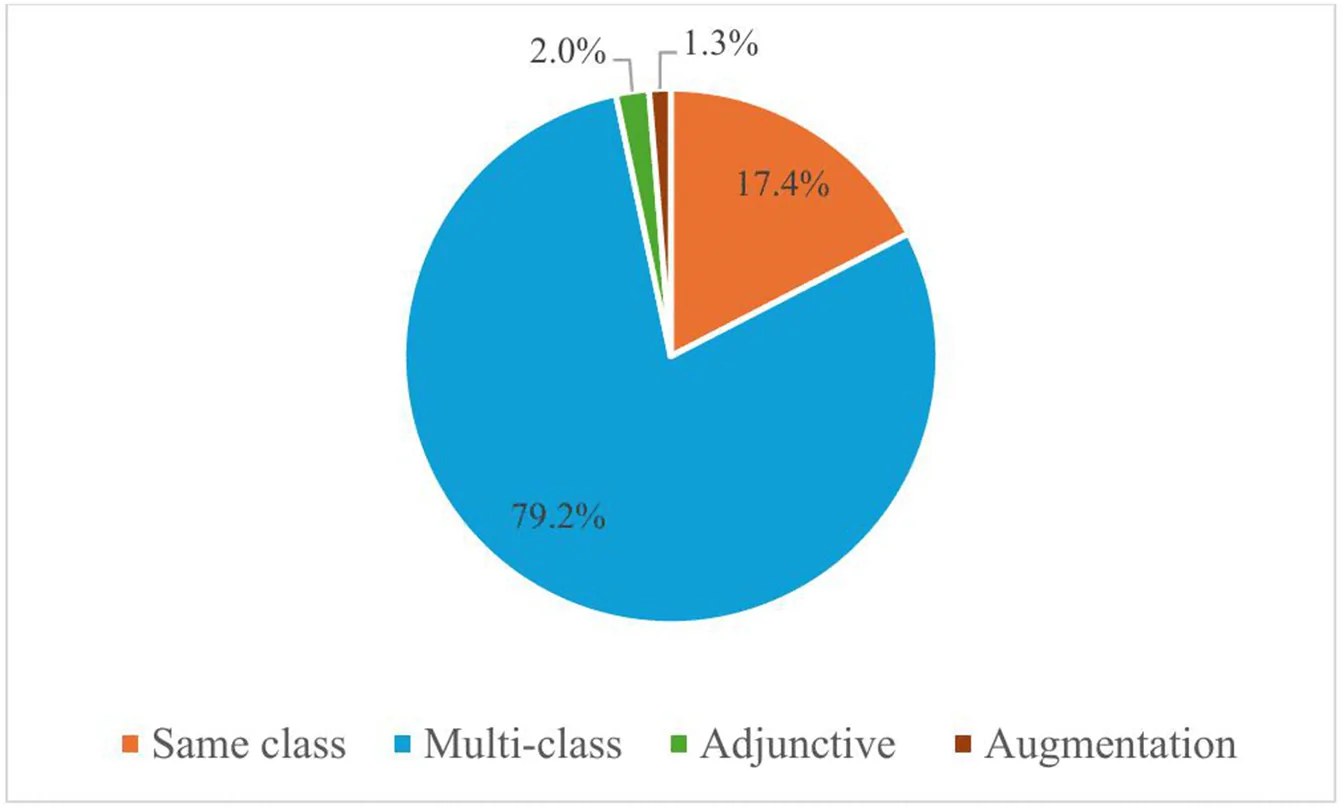
Types of psychiatric polypharmacy among 300 participants in Sbrana Psychiatric Hospital.

Fig 2 displays the frequency of different types of psychiatric polypharmacy (in %). Multi-class polypharmacy was the most common type of psychiatric polypharmacy seen among the participants (79.2%), while same-class polypharmacy was seen in 17.4% of the participants.

### Predictors of psychiatric polypharmacy

The results showed that our model was a good fit, as indicated by the Hosmer-Lemeshow Goodness-of-Fit Test, with a chi-square value of 4.494 and a p-value of 0.810, which is greater than 0.05. The multicollinearity test showed a tolerance greater than 0.1 and a VIF less than 10, indicating no multicollinearity among the independent variables in the multivariable regression model. The full model, containing six independent variables (Table 3), was statistically significant, χ² (6, N = 300) = 64.975, p < 0.001, indicating that our model was able to distinguish respondents with polypharmacy from those without.

**Table 3:** The regression model of the predictors of psychiatric polypharmacy among 300 participants in Sbrana Psychiatric Hospital.

Characteristics	N/n	COR	95% CI		AOR	95% CI		P
			Lower	Upper		Lower	Upper	
**Duration of psychiatric treatment**	**300**	1.62	1.23	2.12	1.63	0.82	3.23	0.162
**Mode of care**	**300**							
Inpatient	150	3.26	2.03	5.22	5.00	2.70	9.25	**< 0.001**
Outpatient	150							
**Substance use disorders**	**300**							
Yes	16	4.46	1.24	16.0	4.60	1.18	17.86	**0.028**
No	284							
**Bipolar mood disorders**	**300**							
Yes	216	2.96	1.74	5.03	2.67	1.49	4.80	**< 0.001**
No	84							
**Previous admission**	**300**							
Yes	199	2.01	1.23	3.28	1.92	0.99	3.71	0.052
No	101							
**Specialist care given**	**300**							
No	173	0.62	0.39	0.98	0.90	0.48	1.67	0.734
Yes	127							

**Significant p-values in bold.**

After a multivariable logistic regression, only the mode of treatment, a diagnosis of bipolar mood disorder, and having a substance use disorder were found to be statistically significant. Participants who were seen as inpatients were more likely to be on psychiatric polypharmacy as compared to those who were seen as outpatients (AOR = 5.00; 95% CI: 2.70,9.25). Those who had a substance use disorder were found to be four times more likely to be on psychiatric polypharmacy than patients who had no substance use disorder (AOR = 4.60; 95% CI: 1.18,17.86). Participants who had a diagnosis of bipolar mood disorder were more likely to be on psychiatric polypharmacy as compared to those without bipolar mood disorder (AOR = 2.67; 95% CI: 1.49,4.80) (Table 3).

## Discussion

We set out to find the prevalence and pattern of psychiatric polypharmacy and to determine the factors associated with psychiatric polypharmacy among adult patients in Sbrana Psychiatric Hospital. The overall prevalence of psychiatric polypharmacy was 49.7%, and the most common type of psychiatric polypharmacy was multi-class polypharmacy at 79.2%. Treatment setting, having a diagnosis of bipolar mood disorder, and having a substance use disorder were the factors found to be significantly associated with psychiatric polypharmacy.

The observed psychiatric polypharmacy prevalence of 49.7% in our study is consistent with the 46.9% reported by Alharbi and colleagues from a study conducted in Saudi Arabia [40] and with the 48% reported in a study conducted in Malaysia [60].

Nonetheless, we found that the prevalence in the current study is higher than that reported in a local study by Olashore and colleagues, which found 29.2% in a different population [57]. Compared with our study population, which included adult participants, the participants in the study by Olashore et al. were children and adolescents and generally had a lower likelihood of psychiatric polypharmacy [57]. The disparity in these findings may suggest that older patients are more likely to receive polypharmacy as compared to younger patients due to cautious prescribing practices in children and adolescents.

Conversely, a prevalence rate higher than that reported in the current study has also been reported in multiple other studies [14,15,21]. In Nigeria, Adeponle and colleagues reported a psychiatric polypharmacy prevalence of 92.0% [21]. One possible explanation for the observed discrepancies in the current research may be attributed to variations in the definition of psychiatric polypharmacy; for instance, the study conducted in Nigeria [21] examined total polypharmacy, which was not a focus of the current study. Total polypharmacy is the total count of medications prescribed and includes non-psychotropic medications [52]; thus, it is reasonable that these studies would have higher rates of polypharmacy than the present study, which excluded total polypharmacy.

Overall, this study shows that adult psychiatric patients in Botswana have higher rates of psychiatric polypharmacy than children and adolescents when looking at the studies conducted locally. Moreover, the identified prevalence of 49.2% in our study aligns with the global estimated rate of 13% to 90% [3], reinforcing the growing reliance on multiple psychotropic medications in psychiatry. The variation in prevalence across studies suggests that prescribing practices are influenced by multiple factors such as age group, treatment settings, and the definitions of psychiatric polypharmacy; hence, future studies should look into the disparity regarding the rate of polypharmacy while adjusting for some of these identified confounders

The National Association of State Mental Health Program Directors’ classification of psychiatric polypharmacy was implemented in our study, and multi-class polypharmacy was the most frequent type among the participants at 79.2%. This finding is corroborated by findings from previous studies [15,17]. However, our study revealed a high prevalence of same-class polypharmacy at 17.4% when compared to other studies; the prevalence was higher than that observed in India, where same-class polypharmacy was found to be 8.1% [16]. Similarly, in Nigeria, lower rates of same-class polypharmacy were reported (5.8%) [21].

Studies indicate that same-class polypharmacy carries a higher risk of adverse effects than other types of polypharmacy [27]. For instance, the use of two antipsychotics increases the risk of extrapyramidal side effects and metabolic disturbances [23,60]. The high rate of same-class polypharmacy in our study may be attributable to clinical practice. In Botswana, long-acting injectable (LAI) FGAs are commonly combined with oral SGAs. Often, in patients with poor adherence, these two antipsychotics are usually started with a long-term plan of stopping the oral antipsychotic; oral medications are used for the first few weeks after LAI initiation while waiting for LAI plasma levels to reach steady state. However, prescriptions are not reviewed, and the oral medication continues beyond the intended period, leading to long-term polypharmacy.

We found that treatment setting, having a substance use disorder, and having a diagnosis of bipolar mood disorder were the factors significantly associated with psychiatric polypharmacy. Although most studies did not examine the relationship between treatment setting and psychiatric polypharmacy, our study found an association between these variables. Participants who were seen as outpatients were less likely to be on psychiatric polypharmacy as compared to those who were seen as inpatients. Our findings align with those reported by a national multicentre study conducted by Costa and colleagues in Brazil [43]. Inpatient care may be a risk factor for psychiatric polypharmacy, possibly due to several reasons. Firstly, the severity of illness in hospitalized patients may lead to the use of multiple medications. Secondly, during acute treatment, patients are usually prescribed multiple medications in an effort to achieve rapid stabilization and reduce the length of hospital stay. Lastly, owing to the high frequency of diagnostic dilemmas that are usually seen among inpatients, clinicians may prescribe a trial of different medications, a factor that may account for polypharmacy in this population. Our findings suggest the need for careful medication review before discharge to minimize unnecessary polypharmacy. To implement this, psychiatrists could review inpatients in a timely manner, before discharge, and medications could be reduced. Furthermore, careful selection of medication tailored to the patient’s constellation of symptoms should be encouraged, as a single medication may be used to address multiple symptoms associated with the patient’s syndrome. A typical example involves the treatment of a psychotic patient exhibiting poor sleep and a propensity to experience extrapyramidal symptoms (EPS) through the administration of a medication that is sedating, efficacious, and possesses a low likelihood of inducing EPS.

Patients who had a substance use disorder were found to be four times more likely to be on psychiatric polypharmacy than patients who had no substance use disorder in the present study. These results concur with many other studies that reported that substance use disorder was a factor that demonstrated a significant association with psychotropic polypharmacy [21,29,35,36]. Substance use emerged as a significant predictor, possibly due to the presence of comorbid psychiatric conditions and the need to treat symptoms related to substance withdrawal; in this case, both instances require different medications [61].

In comparison to our findings, when examining the predictors of psychiatric polypharmacy among women receiving psychiatric treatment in Qatar, Elbakary and colleagues found no association between substance use disorder and polypharmacy [18]. The study had only female participants, and since substance use disorders are more prevalent in males than women [62], it is highly likely that substance use would not be a predictor. A study among participants with schizophrenia by Fontanella et al, in the United States, found that participants who had a substance use disorder were less likely to be on polypharmacy [30]. Reasons for conflicting results remain unclear but could result from differences in the disclosure of substance use by study participants. Nonetheless, to avoid polypharmacy among patients with substance use disorders, psychoeducation and non-pharmacological interventions such as motivational interviewing and individual or group substance use counseling should be prioritized in clinical practice.

With regard to the psychiatric diagnosis, in our study, participants who had a diagnosis of bipolar mood disorder were more likely to be on psychiatric polypharmacy than those without a diagnosis of bipolar mood disorder. Several studies have found similar results [17,18,40,41,43]. The association of polypharmacy with bipolar mood disorder likely reflects the clinical challenge in managing the disorder; patients with bipolar mood disorder often have disrupted sleep patterns, which are sometimes managed by benzodiazepines in addition to the mood stabilizers or psychotic symptoms that require the addition of antipsychotics [8,14].

As opposed to our findings, several other studies found a diagnosis of schizophrenia and psychotic disorder as a predictor of psychiatric polypharmacy [16,21,47], while a few studies reported an association of polypharmacy with anxiety and depression [16,41]. One possible reason for the discrepancy across these studies is that substance use disorder could have confounded the relationship between schizophrenia and other psychotic disorders. For instance, schizophrenia patients with comorbid substance use may be more likely to receive polypharmacy, and substance use disorder was a stronger predictor in this study, which might explain why schizophrenia and other psychotic disorders alone were not significant.

### Limitations

Despite our efforts to ensure the reliability of our findings, certain constraints may have influenced the results. Firstly, the cross-sectional design limits our ability to infer causality between predictors and psychiatric polypharmacy. Secondly, the study had a small sample size and was conducted at a single psychiatric hospital; therefore, the findings cannot be generalized. Thirdly, the instrument used to collect data was not validated in Botswana. Nevertheless, it was designed by the author following an extensive review of the literature. It is essential to note that the study’s cross-sectional design precludes the establishment of a causal relationship. Lastly, unmeasured confounders, such as treatment adherence and prescriber preferences, may have influenced the results. However, it offers valuable insights that emphasize safe prescribing practices and can guide future studies in this area.

## Conclusion

Psychiatric polypharmacy is highly prevalent at Sbrana Psychiatric Hospital, reflecting a global trend and underscoring the need for targeted interventions. Patients who are hospitalized or diagnosed with bipolar disorder or substance use disorder were identified as the primary factors driving multiple medication use. These findings reveal the importance of identifying high-risk patients to facilitate medication reviews aimed at improving overall clinical outcomes. For patients requiring inpatient care, particular attention should be given to when they transition to outpatient settings, where deprescribing might be feasible. While psychiatric polypharmacy may be justified in the management of disorders such as bipolar mood disorder and supported by treatment guidelines, it continues to pose risks such as the occurrence of adverse effects and drug interactions [63]. Hence, clinicians should follow evidence-based guidelines to ensure that each medication has a clear therapeutic intent and that patients are continuously monitored for adverse drug reactions (ADRs).

Multi-class polypharmacy is common and often supported by guidelines [17], however, there is a need to develop and promote institutional protocols to guide the use of multi-class polypharmacy, ensuring its use is evidence-based and clinically justified. In addition, strengthening multidisciplinary collaboration among psychiatrists, pharmacists, and other healthcare providers will be essential to ensuring safer prescribing practices. Our study revealed a high degree of same-class polypharmacy, which has raised concerns about its efficacy, safety, and potential adverse outcomes. The frequent use of medications within the same class raises safety issues, including risks of metabolic and extrapyramidal side effects [27]. As such, this may suggest risk for increased extrapyramidal effects and metabolic complications with APP among adult psychiatric patients in Botswana.

Medication burden may also have implications on treatment adherence. More complex medication regimes and a higher number of daily medications can make treatment more difficult for patients to follow consistently. This is particularly relevant in psychiatric populations, where adherence may already be affected by illness-related factors, cognitive difficulties, adverse effects and limited insight. Consequently, prescribing decisions should consider not only the potential efficacy of adding another medication but also the overall complexity and sustainability of the treatment regimen. Ultimately, training clinicians in rational polypharmacy is crucial for optimizing prescribing practices and reducing patient risks in Botswana

## Supporting information

S1 Data Dataset for psychiatric polypharmacy among adult psychiatric patients in Sbrana Psychiatric Hospital. (SAV)
